# Acute Myocardial Infarction in Patient With Triple Negative Breast Cancer After Paclitaxel Infusion: A Case Report

**DOI:** 10.14740/cr325w

**Published:** 2014-07-20

**Authors:** Christopher Esber, Khadijah Breathett, Taha Sachak, Stephen Moore, Scott M. Lilly

**Affiliations:** a Department of Internal Medicine, The Ohio State University Wexner Medical Center, Columbus, Ohio, USA; b Department of Internal Medicine, Division of Cardiology, The Ohio State University Wexner Medical Center, Columbus, Ohio, USA; c Department of Pathology, The Ohio State University Wexner Medical Center, Columbus, Ohio, USA

**Keywords:** Paclitaxel, Kounis syndrome, Allergic myocardial infarction

## Abstract

A 47-year-old woman with breast cancer suffered progressive chest pain and flushing within 5 minutes of her second exposure to paclitaxel. Her symptoms progressed and she became pulseless. Advanced cardiac life support (ACLS) was initiated, and after a series of chest compressions the cardiac monitor revealed ventricular fibrillation. With ongoing ACLS she was transferred to the emergency department where she regained a pulse. Review of electrocardiogram revealed prominent ST elevation in leads V1, V2 and V3 with reciprocal ST depression. She was transferred urgently to the catheterization laboratory. Angiography revealed a high-grade stenosis in the proximal left anterior descending artery (LAD), and drug-eluting stents were placed without complications. She was then transferred to the floor and shortly thereafter suffered pulseless electrical activity and died despite prolonged attempts at resuscitation. Herein, we describe the development of acute myocardial infarction after paclitaxel administration, discuss potential etiologies and review evidence for an allergic component.

## Introduction

Paclitaxel has been associated with adverse cardiac events, including bradycardia, atrioventricular conduction block, ventricular tachycardia, myocardial ischemia and infarction [1]. Previous cases of chest pain and electrocardiographic changes consistent with acute myocardial infarction have been reported [2-10]. Although thrombosis *in situ* is a potential etiology, others have proposed an “allergic myocardial infarction”. The latter may represent a localized inflammatory-mediated reaction [11]. It is plausible that allergic component underlies the other cases of cardiac ischemia and infarction after administration of paclitaxel, in many patients who had no known cardiovascular risk factors [3-7, 9, 10].

## Case Report

**Figure 1:**
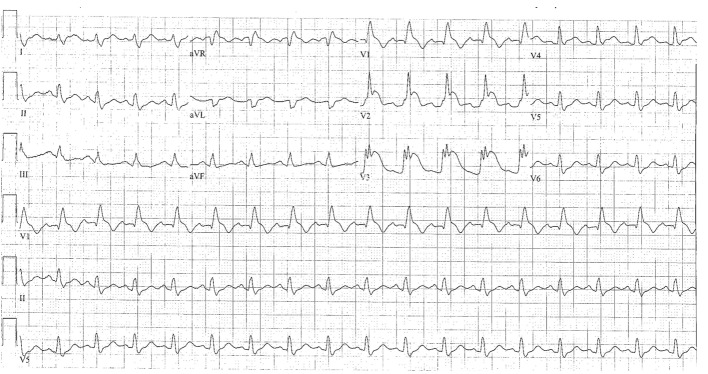
ECG revealing diffuse ST elevations at V1-V3 with reciprocal changes of leads II, III and aVF.

**Figure 2:**
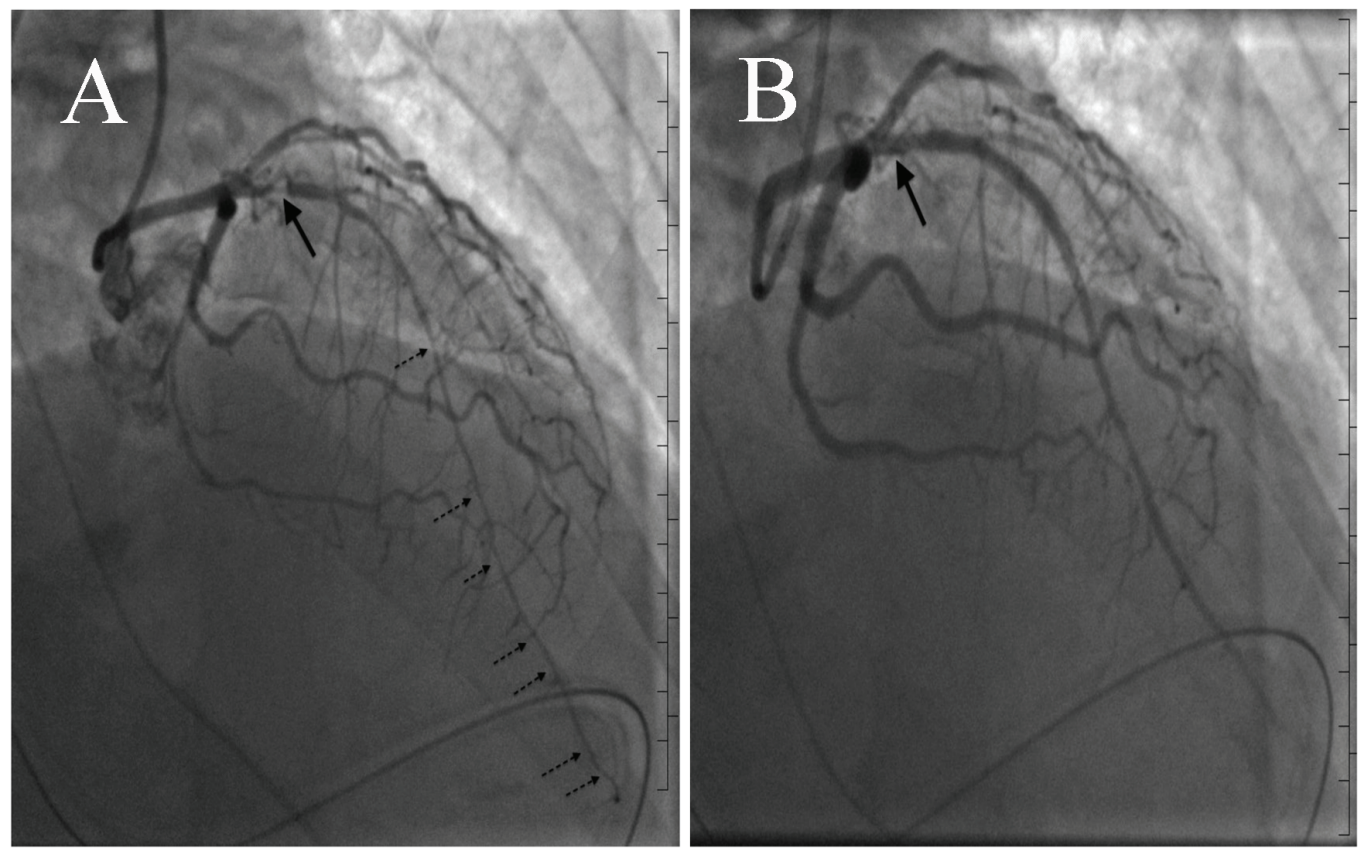
(A) Coronary angiography revealing 95% stenosis of proximal LAD (solid arrow) and diffuse vasospasm distally (broken arrows). (B) Post percutaneous coronary intervention and coronary nitroglycerin (solid arrow).

A 47-year-old female with past medical history of gastroesophageal reflux disease, irritable bowel syndrome, recently diagnosed triple negative breast cancer, and no cardiovascular risk factors, presented to the emergency department undergoing advanced cardiac life support (ACLS). She initially developed chest pain then ventricular fibrillation minutes after starting her second infusion of paclitaxel. Eight days prior to this reaction she had a hypersensitivity reaction to radiotracer used for a technetium-99m scan, which included diffuse hives, pruritus and facial swelling, and required corticosteroids for symptom resolution. On the day she received her second dose of paclitaxel, she experienced facial flushing and complained of chest pain within 5 min of administration. Paclitaxel was stopped abruptly and the patient was given 100 mg of hydrocortisone and 50 mg of IV diphenhydramine. Shortly thereafter she became non-responsive and epinephrine was provided. A cardiac monitor was attached, ventricular fibrillation identified and a shock delivered. ACLS was continued during transportation to the emergency department and an ECG on arrival showed ST elevations in leads V1, V2 and V3 with reciprocal ST depression (Fig. 1). She regained pulse and was taken to the catheterization laboratory, and a high-grade lesion in the proximal left anterior descending artery (LAD) was identified as well as diffuse mid- and distal-LAD vasospasm (Fig. 2A). Two drug-eluting stents (containing zotarolimus) were placed, and intracoronary nitroglycerin was provided. For post procedure there was TIMI-3 flow (Fig. 2B). The patient was transferred to the cardiac intensive care unit in critical condition. Shortly thereafter she experienced another episode of pulseless electrical activity and after multiple rounds of ACLS, she was pronounced dead. Post-mortem examination revealed signs of transmural infarction of the left ventricle and interventricular septum as well as a thrombus within the recently deployed coronary stent.

## Discussion

Paclitaxel is a widely used microtubule-targeted drug for breast, ovarian and non-small-cell lung carcinomas. It is structurally similar to taxane B, a cardiotoxic alkaloid from the yew plant which paclitaxel was derived [3, 4, 12]. Initially, paclitaxel was poorly tolerated due to serious hypersensitivity reactions related to its carrier agent, cremophor EL, polyoxyethylated castor oil, prompting continuous cardiac monitoring [1, 12]. Cardiac toxicity was generally limited to benign arrhythmias in 30% of patients (usually sinus bradycardia) and significant bradyarrhythmia in 0.1% of patients [12, 13]. Fortunately pre-medication protocols with intravenous steroids and anti-histamines were added and reduced many of the adverse effects and the need for continuous cardiac monitoring [1, 12-14]. Over the course of paclitaxel’s use, there have been reports of patients that have developed ischemia or myocardial infarction, but many had significant cardiovascular risk factors including hypertension, smoking history and coronary artery disease, making it challenging to implicate an isolated effect of paclitaxel [1, 12, 15]. However, there have been few other reports where patients developed ischemia or infarction with no known coronary artery disease or significant cardiac risk factors [3-7, 9, 10].

The mechanism of paclitaxel associated with myocardial ischemia is unclear. The case described herein revealed both significant stenosis of the proximal LAD and diffuse LAD vasospasm after administration of paclitaxel. The latter has been a proposed mechanism of paclitaxel-induced ischemia in prior case reports [2-4, 7, 9, 10]. Nguyen-Ho and colleagues postulate that paclitaxel may increase intracellular calcium concentrations, thereby leading to coronary vasospasm [7]. Others proposed the vehicle, cremophor EL, and not paclitaxel itself, is the provocateur by increasing histamine-mediated coronary vasospasm [3, 7, 9]. In our patient, vasospasm superimposed on an obstructive coronary lesion may have been the etiology of the cardiac arrest. Alternatively, rupture of the proximal LAD stenosis may have been the primary insult, as has been suggested in other vascular territories [8, 16].

There also exists evidence of paclitaxel-associated allergic myocardial infarction. In fact, Gemici et al reported a temporal relationship between paclitaxel and myocardial ischemia: the first exposure led to myocardial infarction and subsequent exposure elicited nitroglycerin-responsive chest pain and ST elevations [2]. Pfister and Plice, who presented a case of myocardial infarction during a prolonged reaction to penicillin, first introduced the concept of a medication reaction leading to an acute myocardial infarction in 1950 [17].

The term “allergic myocardial infarct” was introduced shortly thereafter [18], and has been recently extended by Dr. Kounis and colleagues. Kounis syndrome is now believed to be caused by other inflammatory mediators including proteases, tryptase; arachidonic acidproducts; platelet-activating factor; cytokines and chemokines released during the mast-cell activation [19]. Within the cardiology literature, the term “Kounis syndrome” continues to gain popularity as a term which now describes both “allergic angina” and “allergic myocardial infarction” [11, 20]. In addition to natural allergic insults such as insect stings, cases of coronary vasospasm have been reported with ceftriaxone [21], oxaliplatin [22], iodinated contrast media [23], and a chemotherapy regimen of cisplatin and cyclophosphamide [24].

Kounis syndrome has been further classified as having three distinct variants [25]. Type I Kounis syndrome involves coronary vasospasm in patients with previously normal coronary arteries and no predisposing factors for coronary artery disease. Type II Kounis syndrome includes patients with culprit but quiescent pre-existing coronary atherosclerosis in which an acute allergic episode can lead to plaque erosion or rupture and present as an acute myocardial infarction. Type III Kounis syndrome is associated with stent thrombosis of a drug-eluting stent after an allergic reaction and can be verified by pathological evidence of eosinophils on H and E stain and mast cells on Giemsa stain [26]. Our case most closely represents type II Kounis syndrome. However, after placement of two drug-eluting stents coated with the anti-proliferative agent zotarolimus, the patient suffered second cardiac arrest. While it is difficult to determine whether the thrombus seen on post-mortem examination was due to an acute in-stent thrombosis or developed post mortem, after the second myocardial infarction, it is possible that our case also represents type III Kounis syndrome.
